# Self-Driving Laboratories for Development of New Functional Materials and Optimizing Known Reactions

**DOI:** 10.3390/nano11030619

**Published:** 2021-03-02

**Authors:** Mikhail A. Soldatov, Vera V. Butova, Danil Pashkov, Maria A. Butakova, Pavel V. Medvedev, Andrey V. Chernov, Alexander V. Soldatov

**Affiliations:** The Smart Materials Research Institute, Southern Federal University, 178/24 Sladkova, 344090 Rostov-on-Don, Russia; vbutova@sfedu.ru (V.V.B.); pashkov@sfedu.ru (D.P.); mbutakova@sfedu.ru (M.A.B.); pmedvedev@sfedu.ru (P.V.M.); cherno@sfedu.ru (A.V.C.); soldatov@sfedu.ru (A.V.S.)

**Keywords:** self-driving laboratories, artificial intelligence, synthesis, automatization, optimization

## Abstract

Innovations often play an essential role in the acceleration of the new functional materials discovery. The success and applicability of the synthesis results with new chemical compounds and materials largely depend on the previous experience of the researcher himself and the modernity of the equipment used in the laboratory. Artificial intelligence (AI) technologies are the next step in developing the solution for practical problems in science, including the development of new materials. Those technologies go broadly beyond the borders of a computer science branch and give new insights and practical possibilities within the far areas of expertise and chemistry applications. One of the attractive challenges is an automated new functional material synthesis driven by AI. However, while having many years of hands-on experience, chemistry specialists have a vague picture of AI. To strengthen and underline AI’s role in materials discovery, a short introduction is given to the essential technologies, and the machine learning process is explained. After this review, this review summarizes the recent studies of new strategies that help automate and accelerate the development of new functional materials. Moreover, automatized laboratories’ self-driving cycle could benefit from using AI algorithms to optimize new functional nanomaterials’ synthetic routes. Despite the fact that such technologies will shape material science in the nearest future, we note the intelligent use of algorithms and automation is required for novel discoveries.

## 1. Introduction

There is a long history of laboratory synthetic chemistry automation. Such automation designs as continuous flow reactors, single-batch reactors, single-robotic synthesizers, dual-robotic synthesizers, and integrated workstations have already existed for more than three decades. The main objectives of such automatization are to enable the exhaustive experimentation and to increase the productivity and precision of the synthesis [1]. The search for novel unique materials is a crucial step to find fast and effective solutions to several significant challenges that humanity faces now. Conventional high-throughput experimentation strategies for material discovery are already used in modern research laboratories [2]. Automated systems for high-throughput experimentation open a possibility to liberate the scientific workforce, maximize throughput potential, and optimize cost considerations [3]. Moreover, high-throughput screening is already an integral part of pharmaceutical research that brought to the medical practice such drugs as Gefitinib (AstraZeneca), Erlotinib (Roche), Tipranavir (Boehringer Ingelheim), etc. [4]. To go far beyond the standard approaches for human-operated laboratories for chemical synthesis, the novel concept of an AI-driving laboratory has started during recent years.

The pandemic crisis last year has seriously touched in synthetic chemistry workflow, resulting in the problem for the need for a remote-controlled mode of chemical experiments. All the instruments for on-line operation, even for sophisticated flow-chemistry equipment, have only been ready for several years [5], but still these techniques are not very well spread among the chemical community. The ideology of cloud chemistry (i.e., conducting on-line operated chemical experiments using equipment located in someone else’s laboratory in analogy with “cloud computing”) [5] could also result in much more effective use of unique and expensive equipment. At the same time, the speed of artificial intelligence development nowadays takes the form of a revolution almost in all fields of our life, including science and research and development (R&D) sectors [6,7]. Moreover, an AI-driving laboratory could be in operation 24 h a day and 7 days a week, thus the search for novel materials could be done much faster than in a standard human-operated laboratory. Automation of laboratory operation could also minimize the “human factor” as a reason for occasional mistakes. Intrinsically digitized complete protocols of the experiments performed in a fully automated laboratory are also a very important advantage.

## 2. Artificial Intelligence and Machine Learning Role in Materials Discovery

**Figure 1:**
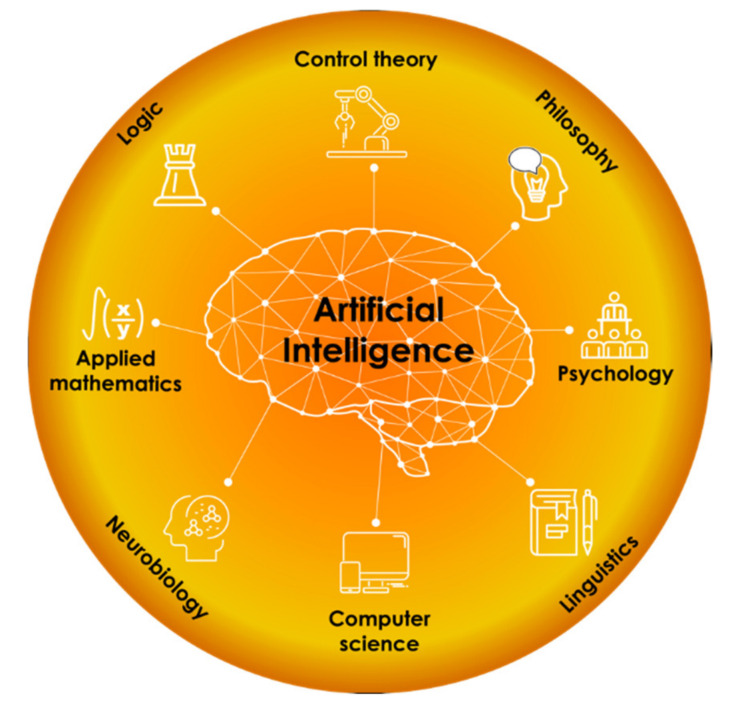
Artificial intelligence (AI) at the “science crossing”.

The field of artificial intelligence (AI) first emerged in the middle of the 20th century. The term “AI” was coined in 1956 at the famous Dartmouth Conference to emphasize the research field dealing with artificial systems that can imitate human-level intelligence. Nowadays, AI is a complicated and multidisciplinary area of expertise and cannot be described only by one theory. In our opinion, it would be better to determine AI’s place as a “crossing” of several science branches, as shown in Figure 1.

Since the moment AI was postulated, its theory and practice constantly spread in various fundamental and applied areas, such as automation, technology, transport, communications, medicine, and entertainment also. During an extended period, until the first decade of the 21st century, in most material science studies, novel results and achievements were supplied by high-performance computer modeling, not AI application. This situation is common because of the existence of well-developed atomistic modeling approaches and software on the one hand, and on the other hand, the training of qualified specialists in the development of new functional materials requires in-depth knowledge in synthetic chemistry, chemical reactivity skills, and a robust understanding of the design and production of novel molecules. These skills take a lot of time, and one can compare them with a kind of art of continuously mastering hands-on practice in the laboratory. However, recent studies in new functional materials discovery tend to be AI filled, and no one can deny that AI methods and approaches turn out to be useless in this area. Nobody can also deny that innovative materials science and chemistry approaches need to be automated and could be more competitive due to AI involvement. We think that AI and machine learning base elements knowledge is an essential aspect for the successful development of modern modeling in chemistry, condensed matter physics, and new functional materials with defined properties.

One of the earliest cases for implementing the imitation of complex natural intelligent systems was the neurobiological approach [8]. This approach supposes that the making of AI systems is unavailable without reproduction material biological sources and processes in living systems. Artificial neural networks (ANNs) in AI are mathematical models [9] and computation tools constructed on principles of simulating the functioning of a natural living organism’s nervous cell network. They work in a parallel computational model comprising many identical processing units, artificial neurons akin to biological neurons. Unlike conventional step-by-step computing algorithms, typical ANN does not use instructions for problem solving. Instead of this, ANN can solve complex nonlinear tasks, making patterns input-output operations by their own “experience learned” with improvements minimizing errors. The largest modern ANN realizations can comprise a layered structure of up to billions of interconnected artificial neurons. Each artificial neuron usually implements some nonlinear weighted function with several input arguments and one output. The function called the activation function behaves like a mathematical “gate,” which is fired when the weighted input sum is over a specified threshold value and the signal is transferred to the next layer or output. Basically, the activation function is modeling the strength of neuron connections like synapses in a biological brain. The most significant problems of ANNs are (1) the initial network topology design and choosing an appropriate math method for neuron activation functions, and (2) neurons weights and biases adjusting. The former problem consists of defining a number of neurons and layers, setting initial connection weights and biases on the one hand, and selecting a theoretical learning strategy for ANN on the other hand. The latter problem is known as the ANNs training task, focusing on defined datasets. Connectionism models, including ANNs, strongly rely on artificial neurons’ dynamic activity, their activation functions, the number of layers, and connection weights. Despite the fact that the leading type of ANNs, deep neural networks [10], can successfully solve today’s scientific problems [11] of classification, such as object recognition and speech processing, while outperforming humans, the connectionism methods and models are inevitably criticized. Until now, many kinds of research has indicated the traditional weakness of the ANN, which lies in the significant difference between the functioning of a biological and an artificial neuron at the physical and mechanistic levels.

It is worth noting that successful ANN application strongly depends on the datasets given, their dimension, and quality, so tasks of the molecule design configuration, functional material properties prediction, and similar problems are not an exception. To properly train ANNs, especially those that use deep learning, significant amounts of labeled data or big data are needed. Big data-driven approaches play a crucial role in the preprocessing steps of collecting, storage, indexing, retrieval, and classification of data in materials science [12]. Digital representation of data retrieved during experiments literally transformed the ways of sharing and exchanging results, supporting information, and software codes by enabling representation and quantification of the discovered material structures. Digitalization and automation have great potential in the field of material synthesis [13] starting from dedicated web portals and specialized databases, such as The Materials Project (https://materialsproject.org (accessed 15 February 2021)), Novel Materials Discovery (https://www.nomad-coe.eu (accessed 15 February 2021)), Materials Platform for Data Science (https://mpds.io (accessed 15 February 2021)), Materials Cloud Archive (https://archive.materialscloud.org (accessed 15 February 2021)), Materials Data Facility (https://www.materialsdatafacility.org (accessed 15 February 2021)), and ending with focusing on flow chemistry robotic platforms [14], automated flow nanomaterial synthesis platforms [15], and problem-oriented specific digital microfluidic devices [16], which will be detailed in the next section.

Machine learning (ML) is an essential part of computational AI and comprises algorithms that are provided to determine unknown dependencies in the data and make machine reasoning with this data by a human-like approach and a so-called learning process. The idea behind ML appeared in 1959, and methods and algorithms belonging to ML develop computational procedures that are implemented in function approximation machines by gaining experience from experiments, observations, measurements, etc. In other words, the main idea of ML is to develop an algorithm that will perform tasks without step-by-step explicit programming, thus the input dataset quality is a crucial point for applying any ML algorithm. The algorithms used in ML use fitting methods, but unlike statistical models based on rigorous mathematical theory, they use iterative approaches to learn from the datasets with the implicit structure by reducing the errors from checking new data for compliance with the previously obtained pattern.

**Figure 2:**
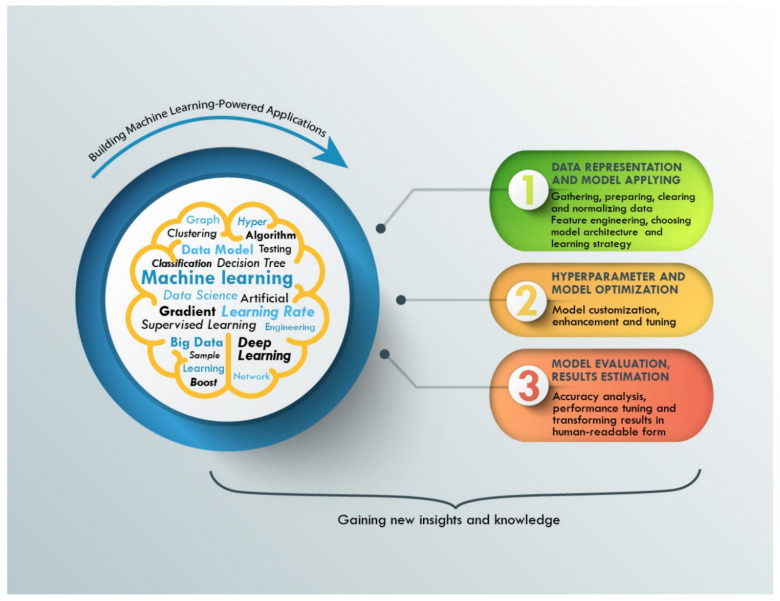
Machine learning powered problem-solving process.

Regardless of the chosen ML method, the process of obtaining a new algorithm for solving a specific problem consists of three components, as shown in Figure 2: (1) data representation and model application; (2) optimization of the model and hyperparameters; and (3) model evaluation and estimation of the results. To achieve better ML application results, we should control the quality of predictions during the training of the ML algorithm procedure. Usually, there is a need for big data and large datasets, because the whole dataset must be split into two parts: (1) a training dataset and (2) validation dataset. The first part is used for training the algorithms and the second part is used for evaluating the quality of predictions. For estimation of the ML model prediction, loss functions are used. In the context of an optimization algorithm, the loss function is a function used to evaluate a chosen model. It returns the value of inaccuracy of the model. The value of the loss function must decrease during the training.

Obtaining a data processing algorithm from the datasets itself can be carried out in various ways. Nowadays, numerous ML approaches have been proposed. For example, ML tasks include solving clustering, classification, and regression problems (Bayesian classifiers, k-nearest neighbors algorithms, decision trees, k-means, and DBSCAN clustering, support vector machines, etc.), pattern recognition using ANNs with different architectures (feed-forward, recurrent, radial basis, long-short term memory, deep belief, deep convolutional, deep residual, generative adversarial networks, etc.), and dimensionality reduction (principal component analysis, multidimensional scaling, t-distributed stochastic neighbor embedding, etc.) while using various training methods (supervised, unsupervised, and reinforcement learning).

There are at least two possible problems in the ML training procedure. The first problem is underfitting. An underfitted model cannot find any dependence in the data, and the accuracy of this model will not be good enough. This situation occurs when we have a small dataset or when we did not make enough iterations during the training procedure. The second situation is overfitting. A model is said to be overfitted when the model gets a lot of data, so it starts learning from the noise and some errors in the training dataset. It leads to excellent quality predictions on the training dataset and poor results on the test dataset. Therefore, we should stop the training procedure when the value of the loss function on the validation dataset begins increasing while that on the training dataset is still decreasing. To estimate the generalization ability of the ML algorithm, the cross-validation procedure and several accuracy metrics are used. The most widely used accuracy metrics for classification problems include area AUC-ROC (area under receiver operating characteristics) [17], F1-score, and metrics for regression problems including MSE (mean squared error) and R2 coefficient of determination.

Within the field of computational materials science, ML becomes powerful instrumentation, providing new abilities both for increased computation model efficiency and for improved prediction of the new material functional properties. As a consequence, this also makes significant progress in the development of a dedicated area within computer science called materials informatics [18]. It should be noted that the problems of material informatics relate not only to the previously considered problems of classification, clustering, and pattern recognition but also to the problems of characterizing and evaluating new properties of materials [19] by predicting and searching for the best estimation ML methods. The problems of the simple application of existing ML approaches and algorithms in the considered area of material informatics are exacerbated due to several existing circumstances. One of their circumstances is the variety of methods and means of carrying out laboratory experiments with substances and materials under many conditions and the resulting variety of datasets with differing accuracy and quality. Another circumstance is the variety of explanations obtained for experiments and models, which must be compared and carefully estimated in order to confirm the plausibility of the hypotheses and patterns put forward. Despite these facts, ML gradually takes positions in the most complex problems, such as HT-DFT calculations [20], atomistic simulations [21], and representing quantum–mechanical interactions [22].

## 3. AI Applications in the Synthesis: From High-Throughput Screening to Machine Learning

**Figure 3:**
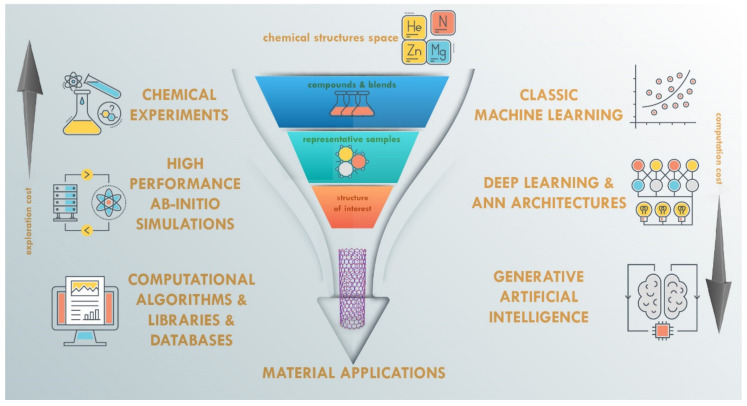
Computerized and AI-equipped approaches in materials discovery.

First of all, we initiate this section with a schematic picture (Figure 3) of the current computerized and AI-equipped approaches in the area of new functional material discovery. Usually, the conventional material discovery process starts from seeking the appropriate set of chemical substances and hands-on laboratory experimentation with blending and composing their part and proportions under various external conditions. As a consequence, these experiments have a rather high cost and take a lot of time for choosing, filtering, and finding the required structural properties. Reducing the impact of this issue can vary in the different ways that can be chosen by the researcher. The left side of Figure 3 shows well-established long applied approaches, but the right side presents the modern AI and deep learning until the perspective of generative AI models. It worth noting that the experimentation cost reduction is usually balanced by the increased computational cost.

Before the age of machine learning advances, researchers had to create hybrid methods for high-throughput screening, adding specific experimental protocols to combinatorial synthesis methods. For instance, resonance-enhanced multiphoton ionization was used for the analysis of solid-state catalyst libraries while searching for solid-state catalysts that activate the dehydrogenation of cyclohexane to benzene. The approach was found to be useful in the study of issues related to the operational lifetimes of catalysts, their resistance to poisoning, their regeneration, and their loss during operation as well.

It was clear even a decade ago that high-throughput technologies have demonstrated their power and they are already on their way to becoming standard technologies or the discovery, development, and optimization of advanced functional materials (for detailed reviews, see [23,24,25]) Genetic algorithms (GAs) and evolutionary strategies were used for years in the field of computational methods applied for the high-throughput search of novel materials. ANNs by themselves and their combination with GA also have a rather long-standing history in high-throughput materials science.

Later research supports the idea that high-throughput computational materials design is a very important emerging area of materials science [23]. This stage of the research and the most important challenges in the field of high-throughput computational materials activity were efficient high-throughput codes, a necessity on open and distributed networks of repositories, the search for fast and effective descriptors, and the development of effective strategies to transfer the knowledge obtained in fundamental science to practical implementations in the high-tech industry.

The Probabilistic Harvard Optimizer Exploring Non-Intuitive Complex Surfaces (Phoenics) algorithm has been developed for both theoretical and experimental chemistry needs. It combines ideas from Bayesian optimization with concepts from Bayesian kernel density estimation, i.e., Bayesian neural networks were used to estimate kernel distributions associated with a particular objective function value from observed parameter points [26]. At each iteration, a surrogate model is constructed, from which new conditions are proposed. The use of this approach makes it possible to formulate an inexpensive acquisition function balancing the explorative and exploitative behavior of the algorithm. It could be applied for the cases where the merit of a set of conditions is evaluated via experimentation or expensive computations, which can possibly be parallelized.

Retrosynthetic analysis is a powerful chemical method. It is based on a step-by-step simplification of the structure of the starting molecule up to simple and accessible starting compounds. The result of the analysis is a scheme of chemical reactions that allow the target compound to be obtained from the available reagents selected during the analysis. The method of combinatorial enumeration of possible options for simplifying the skeleton of a molecule can be carried out using advanced computer simulations, including ANN applications [27]. Two recurrent ANNs were trained on 50,000 experimental reaction examples combining 10 broad reaction types that are commonly used by medicinal chemists. The data-driven neural sequence-to-sequence model proposed can be trained in an end-to-end manner. It was found that it scales more efficiently to larger data sets and naturally incorporates the global molecular environments of the reaction species. Another technique for predicting organic reaction outcomes by using a template-free approach was proposed in [28]. The method exploits the idea of the reaction center pinpointing, thus significantly reducing the number of atoms that one needs to take into account. After direct enumerating of candidate products, the Weisfeiler–Lehman difference network was applied to analyze the high-order interactions between changes occurring at nodes across the molecule under study. A comparison of this approach based on the prediction of a small set of atoms/bonds in the reaction center with a standard template-based approach showed about a 1400 times higher speed.

An interesting concept in continuous flow synthesis is “on-the-fly” reaction optimization [29]. The development of continuous flow self-optimizing reactors could open novel routes for accelerating the progress in several types of complex chemical reactions, like cross-coupling reactions or cyclization reactions, for example. By choosing the right tool for the characterization of the reaction flow (on-line FTIR spectroscopy, for instance), one could relatively easily create a system for self-optimizing reactor systems, even for heterogeneous reactions.

In most cases, a conventional trial-and-error approach has been used to optimize the materials synthesis conditions till now. However, this is a time-consuming as well as a rather costly route, and the optimization efficiency largely depends on the skill and experience of individual researchers. Machine-learning-assisted molecular beam epitaxy was recently successfully applied to synthesize SrRuO3 films, whose quality is sufficiently high to probe their intrinsic quantum transport properties [30]. Three of the most important growth parameters (ruthenium flux rate, growth temperature, and nozzle-to-substrate distance) were optimized by a Bayesian optimization algorithm, which is a sample-efficient approach for global optimization. Each of the three parameters, in turn, was optimized using Bayesian optimization. This technique made it possible to grow the highest quality SrRuO_3_ epitaxial thin films, where the emergence of Weyl fermions could be observed.

For the self-optimization of the system, it is crucial to monitor the properties of products on-fly. For this step, various techniques were applied, such as chromatography, both the high-performance liquid one and gas-liquid, FTIR and fluorescent spectroscopy, and NMR [31]. The other crucial point is effective algorithms for the extraction of available chemical data and automation and control systems [32].

AI and computer algorithms were applied for the optimization of both organic and inorganic synthesis. Perovskites are important inorganic compounds, which attract a lot of scientific interest due to numerous applications. Perovskite ceramics could exhibit ferroelectric or piezoelectric properties, according to composition, and are widely used in modern gadgets. Single-crystal perovskites exhibit outstanding properties, crucial for some applications, for example, for optoelectronics. Moreover, single crystals are of particular interest when screening new materials. Their properties are not affected by grain boundaries or thin-film formation dynamics, and therefore enable the study of fundamental properties of materials. However, the growth of single crystals is a challenging task. Jeffrey Kirman and coauthors applied machine learning and improved robotic synthetics to optimize the antisolvent vapor-assisted crystallization method [33]. In the first step of the experiment, the authors trained the ANN to recognize single crystals in the product and, based on this observation, marked the synthesis conditions as optimal or failed. Then, they varied the concentration of precursors, crystallization times, combinations of solvents, and type of antisolvent. The authors trained an ML model to relate the experimental parameters to the likelihood of success of the crystal growth. Additionally, a UV light-emitting diode was installed in the imaging system to trace the photoluminescence of the obtained crystals. In this way, the high-throughput capabilities were applied to generate a dataset with a relationship between the experimental and chemical parameters with the crystal growth process. Then, this dataset was used to train ML regressors. The authors concluded that they had proposed a new route that could accelerate the discovery of new perovskites.

Vasilios Duros and coauthors reported an optimization of the synthesis of a new polyoxometalate cluster, Na_6_[Mo_120_Ce_6_O_366_H_12_(H_2_O)_78_]·200H_2_O [34]. This gigantic molecule is formed by self-assembly and crystal growth. The authors optimized the synthesis in a workflow that can be followed manually or by a robot. So, they compared the results obtained by a human operator, randomly proposed, and using an active ML algorithm. The last one covered six times more crystallization space than the operator and nine times more than a random search. Moreover, the ML algorithm resulted in a higher crystallization prediction accuracy in comparison with the human experimenters.

Dario Caramelli and coauthors reported chemistry-capable robots that can be networked to coordinate many chemical experiments in real time [35]. This robot is supposed to be a universal tool, and the authors tested it in three fields. Firstly, they applied the proposed system to azo-coupling reactions. Few independent robots carried out experiments and cooperated via the Internet. Each of them mixed two different reagents, and the product was automatically recorded with a webcam, analyzed, and shared in real time using Twitter. Sharing the results and collaboration enabled a reduction of the total number of experiments required to reach the goal. Secondly, the authors investigated a chemical oscillator based on the Belousov–Zhabotinsky reaction to explore the real-time aspect of the networked chemical robots. Each robot got the reactions at different starting points, applied image analysis, and shared data with others. The robots modulated the oscillation period in real time by adding small amounts of starting materials. In this way, they reached identical oscillation periods using the synchronization algorithm. Finally, the authors applied robots to verify the reproducibility of inorganic synthesis using the tungsten polyoxometalate cluster as a model compound. In the first stage, the robots varied the ratio of reagent and pH until it resulted in crystals, monitored by a webcam. These synthesis conditions were marked as optimal and were reproduced 15 times. In total, 13 reaction conditions produced crystals at least once. However, seven of them never produced crystals again. The remaining 6 showed a reproducibility of 11.8–50%.

A lot of reports are focused on the optimization of organic synthesis using ML, and other AI approaches. Part of these works used available databases to train an ANN. For example, Marwin Segler and coauthors applied a Monte Carlo tree search and symbolic AI to discover organic retrosynthetic routes [36]. They compared the proposed system with the traditional computer-aided search method based on extracted rules and hand-designed heuristics. The authors claimed that their system solves more molecules and is 30 times faster than the traditional method. Connor Coley and coauthors used three databases, United States Patent and Trademark Office dataset, Reaxys, and SciFinder databases, to train an ANN [37]. They applied ML and AI to organic synthesis and synthesis planning. Jennifer Wei and coauthors applied ANNs to 16 basic reactions of alkyl halides and alkenes [38]. Using a new reaction fingerprinting method, they succeeded in predicting the reaction types for a given set of reactants and reagents with an accuracy of 85% of test reactions and 80% of selected textbook questions. Moreover, the reported algorithm was further able to guess the product’s structure for a little more than half of the problems.

## 4. Go Beyond: From Microfluidics to a Self-Driving Laboratory

Microfluidic technology is one of the promising methods that opens many routes for creating self-driving devices for high-throughput synthesis of a huge number of advanced nanostructured materials. A successful example of hierarchical MOF nanosheet microcapsules, with precisely controlled sizes, produced on a large scale within minutes with a continuous droplet microfluidic strategy has been presented recently [39]. Aqueous and organic phases were separately injected through a T-junction fabricated as coaxial compositions of two cylindrical channels. The flow of aqueous and organic phases was controlled by syringe pumps. The microdroplets were heated for a polytetrafluoroethylene tube, which was located in a 70 °C water bath. As a result, hierarchical MOF nanosheet microcapsules were obtained and these nanostructures include magnetic and Au nanoparticles encapsulated in and decorated on the capsules, respectively.

Because the bio-medical application of specially designed nano- and micro-particles has became more and more important, nowadays, the emerging interdisciplinary technologies based on microfluidics are taking one of the leading positions in the lab-on-chip field of research and development [40]. Such advanced features of the microfluidic technique as high reproducibility, low batch-to-batch variation, fine control over particle characteristics, and easy to scale-up abilities open bright future perspectives of this method. One should mention the progress of using microfluidics to synthesize nanoparticles with identical core/shell structures, which is very important for the next stages of transferring the technologies from the lab to large-scale production. However, despite the progress in core/shell nanoparticles synthesis and the success of transferring the results to bio-medical research, further work should be focused on unraveling the nature and mechanistic insight into the core/shell nanoparticles formation, which may provide insights for guiding the future nanosystem design, especially in the format of a self-driving lab [40].

To have the ability of synthesizing complex (even core-shell) nanoparticles with arbitrary composition, sizes, and dimensions, one should use both continuous-flow and segmented-flow microfluidic approaches [41]. The continuous-flow microfluidic devices have proven to be an efficient method to mimic the reaction-diffusion environments presented in nature, while segmented-flow microfluidic systems provide, on the contrary, a fast homogenization method. Despite the significant progress made, there are still major hurdles to be overcome in the understanding of reactivity and crystallization at the nanoscale. The engineering of crystalline nanoparticles could, in principle, be done by performing reactions in ultra-small droplets (in the micrometer or sub-micrometer range), which has posed significant experimental challenges till now [41].

Microfluidic technologies have seriously increased the speed and accuracy of synthesis, and simultaneous replaced the human operator with a robotic counterpart. This will undoubtedly open the future route for the next generation of materials science progress [42]. However, current distributed fluidics systems permit <10 steps of a predefined process and are restricted to room temperature and near-atmospheric pressures [42]. Thus, the practicality and applicability of these systems may be limited by the complexity of the synthesis itself. On the other hand, digital microfluidics platforms are highly amenable to miniaturization because they effectively have no moving parts other than the liquid that is to be moved. So, the marriage of robotics and fluidics provides a promising way to enable smart high-throughput synthesis and testing, which could be realized in a self-driving lab concept.

In some automated flow chemistry systems for synthesizing small organic molecules, the synthesis recipes are configured through previously published databases collected with AI, and experimental data obtained during the synthesis is not essential [14]. Although computational screening techniques are a rather effective route for the in silico design of novel advanced materials [43], they could not substitute the traditional “trial and error” of experimentation for the discovery of new materials. It was found that high-throughput computational screening, for example, can eliminate unpromising electrolyte candidates at an early stage and focus on a few promising molecules for experimental synthesis and testing [43]. By using quantum chemical predictions of the electrolyte properties, it is possible to identify promising candidate materials. This will provide useful guidelines for the following synthesis and for the control of some important features of the electrolyte, like stability. These issues, due to reactions with other species or surfaces, as is found in Li−S or Li−O_2_ batteries [43], are more difficult to handle in a pure theoretical high-throughput screening scheme. However, despite these challenges, high-throughput screening in combination with closely coupled experimental investigations can provide important avenues for the design of suitable candidate materials [43].

Microreactors in particular have been shown to have superior heat and mass transfer rates, which is very important for precise control of reaction conditions, especially for the case of exothermic reactions and reactions having energetic intermediates [44]. Integrated automated microreactor systems could incorporate feedback for self-optimization of a chemical reaction. A rather rapid optimization of a Heck reaction using a completely automated microreactor system with feedback control and optimization algorithms has been demonstrated (optimization was obtained only after 19 automated experiments). After obtaining the optimized parameters in a microreactor, the reaction was successfully scaled-up 50-fold in a mesoscale flow reactor.

Continuous flow microfluidic reactors offer a set of advantages compared to the standard batch chemical synthesis mode, including a good heat-transfer rate, and they enable reactions to be performed more safely at elevated temperatures and pressures. The possibility for automation of these reactors has led to the development of self-optimizing systems that use a feedback algorithm coupled with computer process control and integrated on-line analysis [45]. The optimization of the yield of pentyl methyl ether from the reaction of 1-pentanol and dimethyl carbonate in supercritical CO2 was conducted by custom feedback software and without any intervention from the user. It was found that devising a chemical process that needs to be both sustainable and economically viable is not straightforward, as the conditions obtained to be optimal for each specific target feature may be far from optimal for the other.

Because pharmaceutical research (namely drug design) is one of the most funded fields of chemical synthesis, virtual screening has been an important component of modern drug discovery research for years [46]. It was also recognized that both experimental high-throughput screening and computer screening approaches are mutually complementary to each other. Various computational approaches are available at present to complement high-throughput drug discovery technologies. One of the most used virtual screening methods is compound filtering, which is an important complementary technique to high-throughput when it is applied as a front-end technique before experimental high-throughput screening of materials and candidates for medical drugs. Such a pair of techniques could significantly decrease the number of compounds that one needs to test as clinical candidates, a major problem for the pharmaceutical industry at present [46].

To optimize the efforts for high-throughput synthesis of novel materials, it is very important to use specific automation control software, which makes it possible to drive the experiments in a fast and effective way. Chimera [47] is one of these computer codes, which could be used as a part of Phoenics software (Phoenics is an open-source optimization algorithm combining ideas from Bayesian optimization with Bayesian kernel density estimation [26,48]) or as a stand-alone wrapper for other single-objective optimization algorithms. Several tests of Chimera support its applicability for the use as a versatile achievement scalarizing function for multi-objective optimization with costly-to-evaluate objectives. The Chimera wrapper allows the casting of a set of objectives for a number of observations into a single objective value for each observation, enabling single-objective optimization algorithms to solve the multi-objective optimization problem.

Nowadays, there is a growing interest in Internet-based computational chemistry tools and chemical laboratory management on-line platforms. Modern concepts, including Internet of Things (IoT), guide the researcher to the world where every device is connected to the Internet, supplying real-time data exchange between both operating devices and central control cloud computer systems. LeyLab [48] is one of the examples of such an on-line software platform, which consists of four main components: a user-friendly graphical interface; a database where all experiment, equipment, and user data is stored; a user equipment communication module; and a user equipment command module, consisting of code definitions listing commands to be sent to individual equipment [49]. This on-line software platform could deal with several independent experimental parameters and store the data obtained during the reaction process. It should be mentioned as well that this software platform is not limited to only flow chemistry applications but could be used in other useful fields of materials science self-driving laboratory applications.

There are even more effective so-called adaptive design approaches, combining both in silico and real chemical synthesis experiments. The strategy uses inference and global optimization to balance the trade-off between exploitation and exploration of the search space [50]. Such a strategy could significantly accelerate the discovery of an advanced materials process by sequentially identifying the next experiments or calculation parameters to effectively navigate the complex search space. Although there is no universal optimizer for all problems, an adaptive design approach using feedback from an experiment makes it possible to get a very effective procedure for the synthesis of materials with the desired characteristics.

One of the important cases where automation of laboratory research is necessary is the process of co-catalytic reactions’ optimization [51]. This is because the number of possible parameters increases seriously if one should select, for example, two co-catalysts among a large number of possible catalysts and their best relative percentage (concentration). One should also take into account that there are also several other independent parameters of the reaction itself (like temperature, pressure, etc.) to be fitted as well.

**Figure 4:**
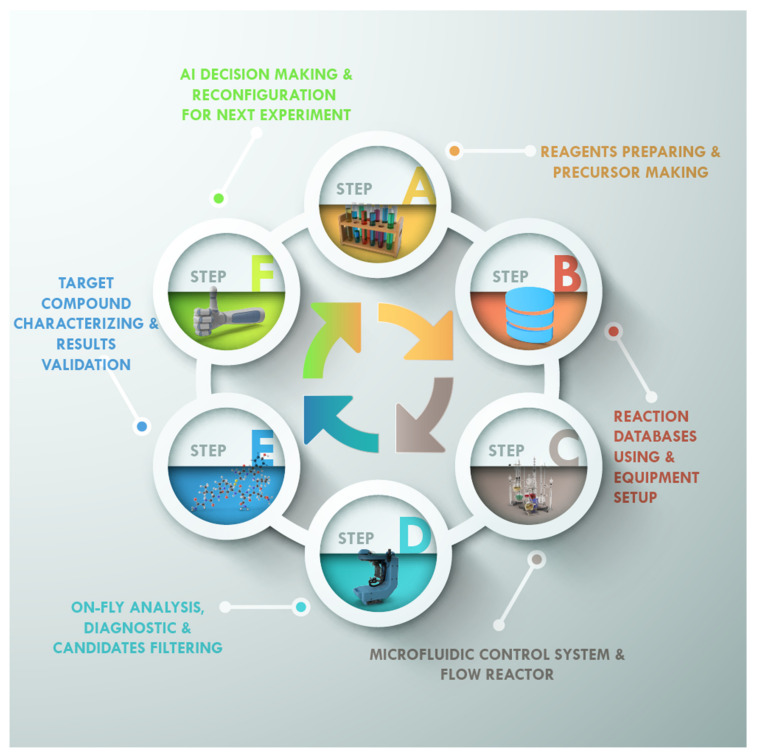
Fully automated closed-loop self-driving laboratory.

A fully automated laboratory combining closed-loop (Figure 4) complex (multi-step) chemical synthesis and several characterization methods with further automatic planning of the next synthesis details makes it possible to increase the speed of the novel materials’ development significantly. An example of a self-driving laboratory for accelerated discovery of organic thin-film materials is to specifically optimize the hole mobility of spiro-OMeTAD, an organic hole transport material common to perovskite solar cells [52]. The platform created (Ada) trains itself to find target parameters without any prior knowledge, enabling iterative experimental designs that maximize the information gain. The automatic platform activity includes the following steps: (1) Robotic preparation of spin coating inks, (2) robotic spin coating of thin-film samples, (3) robotic thermal processing of thin-film samples, (4) robotic dark-field photography, (5) robotic UV-vis-NIR spectroscopy, (6) four-point probe conductance instrumentation and characterization, (7) pseudomobility calculation, and finally, (8) determining the next experiment with Bayesian optimization.

Advances on high-throughput experimentation combined with the self-driving approach for the design of photostable material composites for organic photovoltaics have been demonstrated recently [53]. To go beyond ternary organic photovoltaics systems, one really needs to use automation while performing the synthesis, as there are too many parameters to be varied during the search of the best composition and a large volume of the ingredients should be used as well. It was found that while with conventional approaches, roughly 100 mg of material would be necessary, the robot-based platform can screen 2000 combinations with less than 10 mg, and ML-enabled autonomous experimentation identifies stable compositions with less than 1 mg [53]. Thus, self-driving by an ML lab could not only increase the speed of the materials search significantly, but also make it possible to decrease the volume of starting reagents by two orders of magnitude.

If a mobile robot in the chemical lab is combined with AI technologies, the resulting self-driving lab could solve rather challenging complex tasks, including the search for the optimal photocatalytic system within the process of hydrogen production from water. It was shown that such a system could operate completely autonomously for several days and the scientific results were equivalent to those produced by the human researcher, who should use several months of efforts for such a study to fully explore five hypotheses under the study in the same level of detail using standard manual approaches [54]. The autonomous robot used in the research also requires half a day to set it up initially, but it then runs unattended over multiple days (1000 experiments take only 0.5 days for the robot, while a manual hydrogen evolution measurement requires about 0.5 days of researcher time per single experiment. Hence, the autonomous workflow is 1000 times faster than manual methods and at least 10 times faster than semi-automated but non-autonomous robotic workflows [54].

In the next report, ML and AI were applied to the optimization of the experiment. Brandon J. Reizman and Klavs F. Jensen reported optimizing the alkylation of the 1,2-diaminocyclohexane process in microfluidic conditions [55]. They applied synthesis in microdroplets and varied the solvent, temperature, reaction time, and precursor ratio to generate a high yield of the desired product. The obtained products were quenched and analyzed by on-line LC/MS.

The production rate of a Paal–Knorr reaction was improved within a constrained temperature and residence time design space using in situ infrared spectroscopy to monitor the reaction in microreactors using a multidirectory scheme [56]. A conjugate gradient algorithm showed higher efficiency compared to a set of optimization methods. The compromise between the production rate and conversion was analyzed as well.

A “smart” microreactor system coupled with a fluorescent spectrometer was used to improve the synthesis conditions for CdSe nanoparticles [57]. The measured PL spectra were reduced to a scalar ‘dissatisfaction coefficient’ that was minimized in the course of the synthesis optimization procedure. The effects of the temperature, reaction rate, CdO:Se ratio, and precursors injection rates were analyzed.

Virtual screening of 1.6 million molecules was used to select 400,000 promising candidates for time-dependent density functional theory calculation (TDDFT) [58]. The library was generated using energy (singlet-triplet gap), structure (donor: bridge:acceptor ratio), and technological (molecular mass) considerations. The TDDFT simulations resulted in thousands of promising novel organic light-emitting diode molecules. Selected candidate molecules were synthesized and studied experimentally. The theoretically predicted values showed agreement with experimental data within the known accuracy of TD-DFT and the noise in experimental measurements [58].

A fully reconfigurable continuous-flow chemical synthesis system enabled optimization of a number of chemical reactions [59]. The modular system equipped with reactors and separators, in situ high-performance liquid chromatography, mass spectrometry, and vibrational spectroscopy was controlled by a user-friendly software based on MATLAB and LabVIEW. Six reactions were selected for optimization: Buchwald–Hartwig amination, Horner–Wadsworth–Emmons olefination, reductive amination, Suzuki–Miyaura cross-coupling, nucleophilic aromatic substitution (SNAr), and a visible light photoredox reaction. For each reaction, a four-step protocol was used: design of reaction sequence, loading of the reactants to the system, selection of the parameter boundaries, and starting of the automated optimization using stable noisy optimization by the branch and fit (SNOBFIT) algorithm [60].

The other option is training a neural network on real laboratory experiments and verifying the obtained data with reported results. Jarosław Granda and coauthors presented an organic synthesis robot [61]. The authors claimed that this robot performs chemical reactions and analysis faster than humans. Moreover, it can effectively navigate the reaction space. Based on a small number of experiments, it can successfully predict the reactivity of possible reagent combinations. The developed robot is equipped with such analysis tools as a flow NMR system, a mass spectrometer, and an IR-spectroscopy system. It predicted the reactivity of about 1000 combinations of reagents with an accuracy greater than 80%.

Another interesting example was a materials design using machine learning on the data from failed experiments [62]. The physicochemical properties of organic molecules, atomic properties of inorganic reactants, and experiment reaction parameters were selected as descriptors. Taking into account these data, a support vector machine model was built that reached a 78% accuracy. Inverting the machine learning model for fail synthesis gives new insights for the successful synthesis conditions.

## 5. Conclusions

Recent trends and studies show increasing and spreading AI-powered approaches in materials design. However, we think AI is embracing more and more essential parts of the hands-on work in chemical laboratories penetrating the most complex areas, which previously were not possible without the direct involvement of humans. One of the most attractive possibilities in this way are the self-driving laboratories. The progress in the automatization of laboratory equipment gave rise to the integration of the reagents, manipulations, and on-fly analysis in a single self-driving setup. Such setups make it possible to perform operator-free high-throughput screening or smart synthesis optimization. Moreover, this approach leads to precise control of material characteristics and improved experimental reproducibility. The transition from traditional to operator-free experimentation could change the duties of researchers, liberating them from routine operations. Despite the expectations from AI driving chemical labs that are rather promising, one should keep in mind that it will not be possible to eliminate the necessity of smart human analysis of the results obtained. The challenge for the chemist is not only the use of AI, but also the intelligent use of algorithms and automation for novel discoveries rather than just getting several new molecules [63].

The miniaturization of microfluidic devices as well as smart synthesis optimization will reduce the amount of the reagents for the experimentation. This will minimize the price of the development of new materials and the chemistry footprint in general. Self-driving microfluidic devices have already accelerated the discovery of new materials in a number of pioneering laboratories. We expect that this will approximate the widespread deployment of self-driving laboratories as the introduction of computer simulations in nuclear physics in 1940s made widespread computers’ deployment.

Finally, combining self-driving laboratories with synchrotron radiation facilities could provide even more breakthroughs not only in chemistry, but also in physics, biology, medicine, etc.

## Data Availability

Not applicable.
